# Resistant Hypertension after Hypertensive Intracerebral Hemorrhage Is Associated with More Medical Interventions and Longer Hospital Stays without Affecting Outcome

**DOI:** 10.3389/fneur.2017.00184

**Published:** 2017-05-03

**Authors:** Daojun Hong, Dana Stradling, Cyrus K. Dastur, Yama Akbari, Leonid Groysman, Lama Al-Khoury, Jefferson Chen, Steven L. Small, Wengui Yu

**Affiliations:** ^1^Department of Neurology, University of California at Irvine, Irvine, CA, USA; ^2^Department of Neurology, The First Affiliated Hospital, Nanchang University, Nanchang, Jiangxi, China; ^3^Department of Neurosurgery, University of California at Irvine, Irvine, CA, USA

**Keywords:** intracerebral hemorrhage, resistant hypertension, intensive care unit, length of stay, functional outcome

## Abstract

**Background:**

Hypertension (HTN) is the most common cause of spontaneous intracerebral hemorrhage (ICH). The aim of this study is to investigate the role of resistant HTN in patients with ICH.

**Methods and results:**

We conducted a retrospective study of all consecutive ICH admissions at our medical center from November 2013 to October 2015. The clinical features of patients with resistant HTN (requiring four or more antihypertensive agents to keep systolic blood pressure <140 mm Hg) were compared with those with responsive HTN (requiring three or fewer agents). Of the 152 patients with hypertensive ICH, 48 (31.6%) had resistant HTN. Resistant HTN was independently associated with higher body mass index and proteinuria. Compared to the responsive group, patients with resistant HTN had higher initial blood pressures and greater requirement for ventilator support, hematoma evacuation, hypertonic saline therapy, and nicardipine infusion. Resistant HTN increases length of stay (LOS) in the intensive care unit (ICU) (4.2 vs 2.1 days; *p* = 0.007) and in the hospital (11.5 vs 7.0 days; *p* = 0.003). Multivariate regression analysis showed that the rate of systolic blood pressure >140 mm Hg and duration of nicardipine infusion were independently associated with LOS in the ICU. There was no significant difference in hematoma expansion and functional outcome at hospital discharge between the two groups.

**Conclusion:**

Resistant HTN in patients with ICH is associated with more medical interventions and longer LOS without effecting outcome at hospital discharge.

## Introduction

Uncontrolled hypertension (HTN) is the most common cause of spontaneous intracerebral hemorrhage (ICH) (1, 2). Hypertensive ICH is a type of stroke with intraparenchymal bleeding from hypertensive damage to blood vessel walls. Chronic HTN produces microangiopathy characterized by lipohyalinosis, fibrinoid necrosis, and development of Charcot–Bouchard aneurysms, affecting penetrating arteries throughout the brain. The predilection sites for hypertensive ICH include the basal ganglia (40–50%), lobar regions (20–50%), thalamus (10–15%), pons (5–12%), cerebellum (5–10%), and other brainstem sites (1–5%) (3, 4).

Data from numerous studies have identified high systolic blood pressure (SBP) as the major risk factor for ICH (5–7). SBP variability predicts poor outcome and neurological deterioration (8–10).

Two large randomized controlled trials have demonstrated that early intensive lowering of SBP to less than 140 mm Hg is safe without significant outcome impact (11, 12). Acute lowering of SBP to 140 mm Hg has been recommended for the management of spontaneous ICH (13).

However, it is challenging to promptly and effectively control SBP in patients at risk for resistant HTN (14–17). Resistant HTN is defined as blood pressure that remains above normal despite of concurrent use of three antihypertensive agents of different classes (15, 16). Data derived from cross-sectional studies and *post hoc* analyses of clinical trials have estimated the prevalence of resistant HTN to be about 8–15% of all patients being treated for HTN (17, 18). Currently, little is known about resistant HTN in patients with ICH. We sought to evaluate the potential role of resistant HTN in ICH.

## Materials and Methods

### Patients

All consecutive ICH admissions at the University of California, Irvine Medical Center between November 2013 and October 2015 were reviewed retrospectively. The patient list was compiled by searching the electronic medical record using International Classification of Diseases Ninth Revision code 431 and by stroke center primary data collection for the AHA *Getting with the Guidelines registry*. We then conducted extensive chart review to identify those with hypertensive ICH.

### Study Protocol

**Figure 1:**
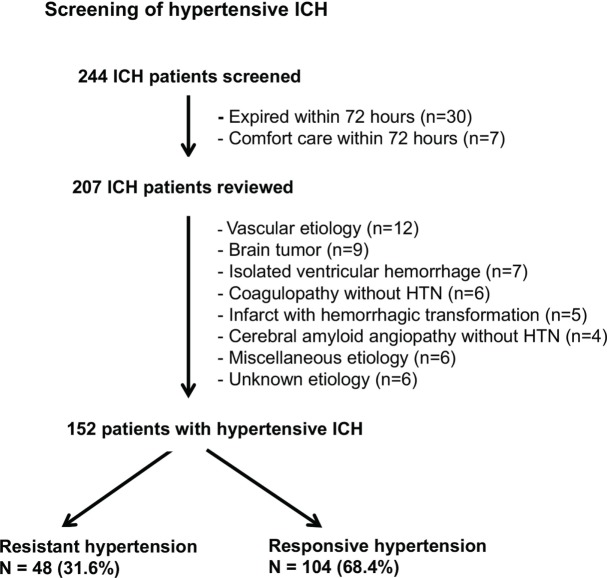
**Screening flowchart for patients with hypertensive intracerebral hemorrhage (ICH)**.

The screening of hypertensive ICH is described in Figure 1. Exclusion criteria included death or comfort care within 72 h of admission, all non-hypertensive ICH, isolated intraventricular hemorrhage, and ICH with subdural hemorrhage. Non-hypertensive ICH was defined as primary ICH without clinical evidence of HTN during the hospital stay. Patients with both HTN and cerebral amyloid angiopathy (CAA) or coagulopathy were counted as hypertensive ICH.

Hypertensive ICH patients were divided into two groups: patients on four or more antihypertensive agents at discharge (resistant group) and patients on three or fewer antihypertensive agents (responsive group) per AHA guidelines (15).

The following demographics and medical history were collected for patients with hypertensive ICH: age, gender, race, body mass index (BMI), social history, medical history, and home medications (antiplatelet, anticoagulant, and antihypertensive agents). Clinical data collected were initial SBP and diastolic blood pressure, Glasgow coma scale score, hyponatremia, serum creatinine, urine protein, ICH location, ICH volume, midline shift, ICH score, ICH volume increase within 24 h, fever (>38°C), pneumonia or other infections, deep vein thrombosis (DVT), seizure activities, rate of SBP > 140 mm Hg during the intensive care unit (ICU) stay, ventilator support, external ventricular drainage (EVD), hematoma evacuation, use of hypertonic saline, duration of nicardipine infusion, length of stay (LOS) in the ICU and the hospital, disposition, number of oral antihypertensive agents, and modified Rankin Scale (mRS) score at discharge.

The ICH volume was calculated by the ABC/2 formula (18). The ICH score was estimated as previously described (19). ICH location was classified as deep (basal ganglia or thalamus), lobar, brain stem, or cerebellar. Midline shift was measured at the level of the septum pellucidum on CT scan. The outcome at discharge was divided into favorable functional recovery with mRS scores 0–3 and unfavorable functional recovery with mRS scores 4–6. Disposition was classified as self-destination (home or acute rehabilitation facility), dependent destination (acute care facility or skilled nursing facility), or death.

### Statistical Analysis

Data analyses were performed using SPSS software. Categorical or dichotomous variables were expressed as frequency distribution and percentages. Group comparisons of variables were performed using the chi-square test, Fisher exact test, Student’s *t*-test, or Mann–Whitney test as appropriate. Correlation analyses between variables were evaluated using Pearson’s correlation coefficient. Multivariate linear regression was performed to assess the independent effect of variables. A *p* value of 0.05 or less was considered significant.

## Results

### Resistant HTN in Patients with ICH

Of the 244 ICH admissions, 152 patients met the inclusion criteria for hypertensive ICH and 92 patients were excluded due to early death, non-hypertensive causes, or unknown etiology (Figure 1). There were 48 patients (31.6%) in the resistant group and 104 (68.4%) in the responsive group.

### Clinical Characteristics of Patients with Resistant and Responsive HTN

The baseline characteristics of the two study groups are shown in Table 1. The patients with resistant HTN were associated with younger age, obstructive sleep apnea, chronic renal failure, higher initial blood pressures, increased serum creatinine, and proteinuria. There was no significant difference between the two groups in home use of antithrombotic and antihypertensive medications, ICH volume, location, or severity. After adjustment for age, race, and gender, logistic regression analysis showed that BMI (odds ratio, 1.073; 95% confidence interval, 1.005–1.145; *p* = 0.034) and proteinuria (odds ratio, 3.204; 95% confidence interval, 1.440–7.131; *p* = 0.004) were independently associated with resistant HTN.

**Table 1:** **Clinical characteristics of ICH patients with resistant or responsive HTN**.

Variables	Resistant group (*n* = 48)	Responsive group (*n* = 104)	*p* Value
Age (years)	58.0 (52.0, 67.8)	63.0 (51.0, 76.8)	0.041*
Male	30 (62.5)	63 (60.6)	0.821
Race			0.445
Hispanic American	22 (45.8)	48 (46.2)	
Non-Hispanic American	12 (25.0)	29 (27.9)	
Asian-American	9 (18.8)	23 (22.1)	
African-American	5 (10.4)	4 (3.8)	
Smoking	7 (14.6)	16 (15.4)	0.930
Alcohol use	8 (16.7)	15 (14.4)	0.720
Diabetes	16 (33.3)	32 (30.8)	0.752
Hyperlipidemia	19 (39.6)	40 (38.5)	0.895
Cardiovascular events	11 (22.9)	13 (12.5)	0.102
OSA	8 (16.7)	6 (5.8)	0.039*
Chronic renal failure	13 (27.1)	10 (9.6)	0.005*
Use of antiplatelet agents	11 (22.9)	23 (22.1)	0.912
Use of anticoagulation agents	4 (8.3)	10 (9.6)	1.000
Use of anti-HTN medications	25 (52.1)	44 (42.3)	0.260
Initial GCS			0.501
9–15	38 (79.2)	87 (83.7)	
3–8	10 (20.8)	17 (16.3)	
Initial SBP (mm Hg) in ED	201.5 (186.5, 223.8)	180.0 (157.3, 200.0)	0.001*
Initial DBP (mm Hg) in ED	99.5 (90.0, 123.0)	95.0 (79.0, 106.8)	0.016*
BMI (kg/m^2^)	27.8 (24.1, 33.2)	26.0 (23.0, 29.7)	0.010*
Serum creatinine (mg/dL)	1.0 (0.7, 1.5)	0.8 (0.6, 1.0)	0.008*
Proteinuria	28 (58.3)	35 (33.7)	0.004*
Initial ICH volume (mL)	17.4 (5.5, 32.1)	11.8 (4.7, 21.6)	0.055
Midline shift (mm)	0.0 (0.0, 4.9)	0.0 (0.0, 3.0)	0.105
ICH location			0.581
Deep	28 (58.3)	69 (66.3)	
Lobar	14 (29.2)	21 (20.3)	
Brain stem	4 (8.3)	7 (6.7)	
Cerebellum	2(4.2)	7 (6.7)	
ICH score			0.486
0–2	41 (85.4)	84 (80.8)	
3–5	7 (14.6)	20 (19.2)	

*BMI, body mass index; DBP, diastolic blood pressure; ED, emergency department; GCS, Glasgow coma scale; ICH, intracerebral hemorrhage; IQR, interquartile range; OSA, obstructive sleep apnea; SBP, systolic blood pressure; HTN, hypertension*.

*Variables are presented as *n* (%) in nominal data or median (IQR) in continuous data*.

*p < 0.05.

### Antihypertensive Therapy

Patients with SBP > 140 mm Hg were initially treated with labetalol or hydralazine 10 mg iv *pro re nata* and nicardipine infusion 2.5–15 mg/h. The intravenous medications were then quickly transitioned to oral antihypertensive agents, starting in the ICU. The commonly used oral antihypertensive agents included calcium channel blocker (CCB) amlodipine, angiotensin-converting enzyme inhibitor (ACE-I) lisinopril or benazepril, angiotensin II receptor blocker (ARB) losartan, diuretics hydrochlorothiazide or spironolactone, β-blocker metoprolol, α/β-blocker labetalol or carvedilol, central α agonist clonidine, and vasodilator hydralazine. The oral antihypertensive agents taken by patients at hospital discharge are shown in Table 2. Approximately 29.2% (14/48) of patients with resistant HTN needed five or more antihypertensive agents. Patients with resistant HTN were more likely to be taking CCB, ACE-I, α/β blocker, diuretics, vasodilator, and central α-agonist than the responsive group.

**Table 2:** **The use of antihypertensive agents in resistant and responsive groups**.

Antihypertensive agents	Resistant group (*n* = 48)	Responsive group (*n* = 104)	*p* Value
CCB	42 (87.5)	61 (58.7)	0.001*
ACE-I	40 (83.3)	68 (65.4)	0.023*
α/β Blocker	32 (66.7)	37 (35.6)	0.001*
Diuretics	23 (47.9)	11 (10.6)	0.001*
β Blocker	15 (31.3)	25 (20.0)	0.348
Vasodilator	31 (64.6)	8 (7.7)	0.001*
Central α-agonist	13 (27.1)	6 (5.8)	0.001*
ARB	8 (16.7)	11 (10.6)	0.291

*CCB, calcium channel blocker; ACE-I, angiotensin-converting enzyme inhibitor; ARB, angiotensin II receptor blocker*.

*p < 0.05.

### Medical Complications, Interventions, and Outcome

As shown in Table 3, patients with resistant HTN were more likely to have fever, hyponatremia, and non-respiratory infections than those with responsive HTN. Despite higher initial blood pressures (Table 1) and a higher rate of SBP > 140 mm Hg (37.2 vs 25.2%; *p* = 0.001) (Table 3) in the resistant group, there was no significant difference in average increase of the ICH volume at 24 h (3 ± 6.3 vs 4 ± 3.8). There was no difference in pneumonia, seizure activities, or DVT between the two groups.

**Table 3:** **Clinical features and outcome of resistant and responsive groups**.

Variables	Resistant group (*n* = 48)	Responsive group (*n* = 104)	*p* Value	OR or AD (95% CI)
Fever	20 (41.7)	24 (23.1)	0.019*	2.38 (1.14, 4.96)
Hyponatremia	7 (14.6)	4 (3.8)	0.018*	4.27 (1.19, 15.37)
Pneumonia	10 (20.8)	12 (11.5)	0.130	2.02 (0.80, 5.07)
Other infections	12 (25.0)	12 (11.5)	0.034*	2.56 (1.05, 6.21)
Deep vein thrombosis	2 (4.2)	4 (3.8)	1.000	1.09 (0.19, 6.15)
Seizure	2 (4.2)	6 (5.8)	1.000	0.71 (0.14, 3.65)
ICH volume increase at 24 h	3 (6.3)	4 (3.8)	0.679	1.67 (0.36, 7.76)
External ventricular drainage	7 (14.6)	12 (11.5)	0.598	1.31 (0.48, 3.57)
Ventilator support	23 (47.9)	24 (23.1)	0.002*	3.07 (1.48, 6.35)
Use of hypertonic saline	14 (29.2)	14 (13.5)	0.020*	2.65 (1.14, 6.13)
Hematoma evacuation	9 (18.8)	7 (6.7)	0.025*	3.20 (1.11, 9.19)
Rate of SBP > 140/SBP < 140	37.2 (27.5, 53.5)	25.2 (15.9, 36.1)	0.001*	12.93 (7.97, 17.89)
Rate of DBP > 90/DBP < 90	2.5 (0.5, 5.3)	1.6 (0.0, 4.2)	0.048*	1.58 (−0.73, 3.89)
Duration of nicardipine drip (h)	81.8 (41.0, 172.9)	59.0 (32.8, 111.6)	0.039*	38.90 (6.52, 71.28)
LOS in the ICU (days)	4.2 (1.9, 9.4)	2.1 (1.3, 4.7)	0.007*	2.30 (0.42, 4.19)
LOS in the hospital (days)	11.5 (6.3, 16.8)	7.0 (5.0, 11.0)	0.003*	3.77 (0.02, 7.52)
SBP at discharge (mm Hg)	134.5 (125.3, 146.0)	131.0 (119.0, 137.8)	0.003*	7.32 (2.45, 12.20)
DBP at discharge (mm Hg)	70.5 (61.3, 79.5)	69.0 (61.0, 78.5)	0.567	1.13 (−2.76, 5.02)
mRS at discharge			0.559	0.81 (0.40, 1.65)
0–3	17 (35.4)	42 (40.4)		
4–6	31 (64.6)	62 (59.6)		
Disposition			0.825	
Self-destination	22 (45.8)	53 (51.0)		
Dependent destination	22 (45.8)	44 (42.3)		
Death	4 (8.4)	7 (6.7)		

*AD, absolute difference; CI, confidence interval; DBP, diastolic blood pressure; LOS, length of stay; ICH, intracerebral hemorrhage; ICU, intensive care unit; IQR, interquartile range; mRS, modified Rankin scale; OR, odds ratio; SBP, systolic blood pressure*.

*Variables are presented as *n* (%) in nominal data or median (IQR) in continuous data*.

*p < 0.05.

More patients with resistant HTN required intensive interventions such as ventilator support, hypertonic saline therapy, hematoma evacuation, and nicardipine infusion than the responsive group (Table 3).

The resistant group also had significantly longer LOS in the ICU (4.2 vs 2.1 days; *p* = 0.007) and hospital (11.5 vs 7.0 days; *p* = 0.003) than that of the responsive group.

Multivariate linear regression analysis showed that fever (β coefficient = −0.306; *t* = −3.908; *p* = 0.001), ventilator support (β coefficient = −0.280; *t* = −3.840; *p* = 0.001), and hematoma evacuation (β coefficient = −0.126; *t* = −2.042; *p* = 0.043) were associated with longer LOS in the ICU. After risk adjustment for fever, hyponatremia, infections, ICH score, midline shift, ventilator support, and hematoma evacuation, the rate of SBP > 140 mm Hg (β coefficient = 0.267; *t* = 4.536; *p* = 0.001) and the duration of nicardipine infusion (β coefficient = 0.418; *t* = 7.040; *p* = 0.001) were shown to be independently associated with LOS in the ICU.

There was no significant difference between the two groups in functional outcome (odds ratio, 0.81; 95% confidence interval, 0.40–1.65; *p* = 0.559) and disposition at hospital discharge (*p* = 0.825).

## Discussion

In this single-center cohort, resistant HTN was found in 31.6% patients with hypertensive ICH. It was independently associated with higher BMI and proteinuria. Higher BMI might be a risk factor for resistant HTN, while proteinuria likely reflects end organ damage from chronic HTN. Of note, resistant HTN is seen in only 8–15% of the general hypertensive population (16, 17). There appears to be a higher prevalence of resistant HTN in patients with ICH than in the general hypertensive population. Of note, majority of our ICH patients were Hispanic and Asian. Some of them had never seen a doctor in the past. That may explain the higher prevalence of resistant HTN in our ICH population. There were also significant numbers of patients with lobar or cerebellar hemorrhage in our cohort. Although most of these patients were young and unlikely to have CAA, we cannot rule out the possibility of underlying CAA in some patients.

Medical complications such as fever and infections were reported to increase LOS after ICH (20, 21). In the current study, we demonstrated that patients with resistant HTN had higher rates of medical complications, greater requirement for intensive interventions in the ICU, and longer LOS in the ICU and hospital than patients with responsive HTN. After risk adjustment, the rates of SBP > 140 mm Hg and the duration of nicardipine infusion were independently associated with LOS in the ICU. With intensive BP lowering in both groups, there was no significant difference in average increase of the ICH volume at 24 h and functional outcome at hospital discharge.

Our preliminary findings have a number of implications. First, given higher prevalence of resistant HTN in patients with ICH than in the general hypertensive population, resistant HTN may be a significant risk factor for ICH and should be treated aggressively in the outpatient setting.

Second, 68.4% of hypertensive ICH patients had responsive HTN, and approximately 60% of these patients did not require intensive interventions such as EVD, hematoma evacuation, or ventilation support (Table 3). These findings support the view that patients with minor ICH can be safely monitored in the step-down unit (22–24).

Third, longer LOS in the ICU is associated with significantly higher health-care costs and burdens on patients and their families (25, 26). In view of the lack of outcome benefit from aggressive medical and surgical interventions (11, 12, 27–29), it appears that now it is the time to assess resource utilization in patients with hypertensive ICH. Since higher rates of SBP and prolonged duration of nicardipine infusion were associated with longer LOS in the ICU, early transition from intravenous nicardipine infusion to oral antihypertensive agents in the ICU may potentially reduce LOS and health-care costs.

**Figure 2:**
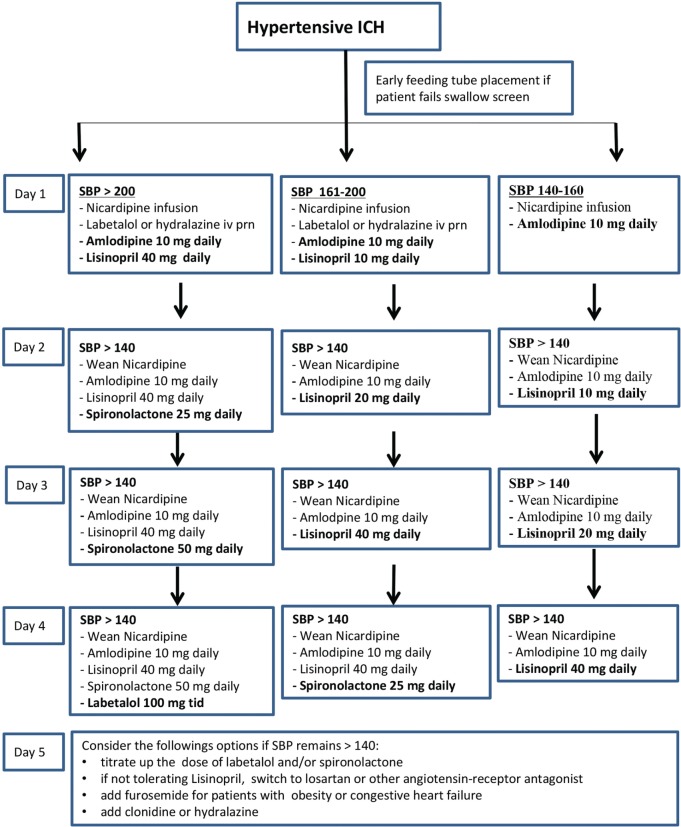
**Oral antihypertensive titration protocol for hypertensive intracerebral hemorrhage (ICH)**.

Finally, little is known about effective management of resistant HTN after ICH. CCB and ACE-I or ARB are widely accepted as first- and second-line drugs for resistant HTN (14). However, the choice of third- and fourth-line antihypertensive agents varies greatly in the management of hypertensive ICH. Hydrochlorothiazide may not be appropriate for patients with large ICH due to the risk of hyponatremia and worsening perihematoma edema. In a recent randomized trial, spironolactone was shown to be very effective in patients with resistant HTN (30). We have successfully used spironolactone and α/β-blocker labetalol as the third- and fourth-line oral agents to control resistant HTN and to wean off nicardipine infusion promptly. A proposed oral antihypertensive titration protocol is shown in Figure 2.

Our study has limitations. First, it was retrospective in nature, and resistant HTN was defined after ICH in the acute care setting. In the acute phase, patients with ICH may have significantly elevated BP due to a stress response. However, stress-induced reactive HTN usually responds to antihypertensive therapy promptly and is unlikely to require more than three oral agents for BP control. Second, given small sample size, the study is insufficiently powered to show significant outcome differences between resistant and responsive groups. The lack of 90-day outcome data made it impossible to know the long-term impact of resistant HTN on functional recovery. A larger sample size study with 90-day outcome data is needed to address the issue.

In summary, resistant HTN may be more prevalent in patients with ICH than in the general hypertensive population. Our data show that resistant HTN increases intensive interventions and LOS in the ICU without significant impact on short-term functional outcome at hospital discharge.

## Ethics Statement

The study was approved by the Institutional Review Board of the University of California, Irvine.

## Author Contributions

DH: study design, data acquisition, analysis, interpretation, and drafting the manuscript. DS: acquisition of data and analysis. CD, YA, and LG: data interpretation and critical revision of the manuscript for important intellectual content. LA-K, JC, and SS: critical revision of the manuscript for important intellectual content. WY: study concept, design, data analysis, interpretation, and manuscript revision.

## Conflict of Interest Statement

The research was conducted in the absence of any commercial or financial relationships that could be construed as a potential conflict of interest. SS serves as editor-in-chief of the international journal Brain and Language and receives compensation from Elsevier Publishing for this service. He is also the recipient of funding from the National Institutes of Health under grants R13 DC011445, P01 HD040605, and P50 MH096889. WY serves as editorial board member for Frontier in Stroke, Stroke and Vascular Neurology and is scientific consultant for Stryker Neurovascular. Other coauthors report no disclosures.
